# Hymenoptera Complex Associated with *Myzus persicae* and *Hyalopterus* spp. in Peach Orchards in Northeastern Spain and Prospects for Biological Control of Aphids

**DOI:** 10.3390/insects10040109

**Published:** 2019-04-16

**Authors:** Yahana Aparicio, Rosa Gabarra, Jordi Riudavets, Petr Starý, Željko Tomanović, Korana Kocić, Juli Pujade Villar, Mar Ferrer Suay, Victor Cuesta Porta, Judit Arnó

**Affiliations:** 1 Institut de Recerca i Tecnologia Agroalimentàries (IRTA), Ctra de Cabrils Km 2, 08348 Cabrils, Barcelona, Spain; yahana_adm@hotmail.com (Y.A.); rosa.gabarra@irta.cat (R.G.); jordi.riudavets@irta.cat (J.R.); 2 Laboratory of Aphidology, Institute of Entomology, Biology Centre, AVCR, Branisovska 31, 37005 České Budějovice, Czech Republic; stary@entu.cas.cz; 3 Institute of Zoology, Faculty of Biology, University of Belgrade, Studentski trg 16, 11000 Belgrade, Serbia; ztoman@bio.bg.ac.rs (Ž.T.); korana.kocic@bio.bg.ac.rs (K.K.); 4 Departament de Biologia Animal, Facultat de Biologia, Universitat de Barcelona, Avda. Diagonal 645, 08028 Barcelona, Spain; jpujade@ub.edu (J.P.V); victor93@gmail.com (V.C.P.); 5 Departament de Zoologia, Facultat de Ciències Biològiques, Universitat de València, Campus de Burjassot-Paterna, Dr. Moliner 50, 46100 Burjassot (València), Spain; mar.ferrer.suay@gmail.com

**Keywords:** Aphididae, Aphidiinae, hyperparasitoids, IPM, conservation

## Abstract

Aphids are a serious pest for peach crops. They have traditionally been managed with insecticides, but there is increasing concern about the risk that insecticides pose to both humans and the environment. As a first step to use biological control in aphid management, we conducted a 3-year field survey in northeastern Spain to determine which parasitoids and hyperparasitoids were most prevalent on two aphids, *Myzus persicae* (Sulzer) and *Hyalopterus* spp. Koch, the most harmful to peach trees. We collected 11 parasitoid species from *M. persicae,* with *Aphidius matricariae* (Haliday) being the most abundant. Two parasitoid species were also collected from *Hyalopterus* spp., *Aphidius transcaspicus* Telenga and *Praon volucre* (Haliday). Hyperparasitoid species overlapped between these aphids but their relative abundances differed. We also discuss the possible impacts of hyperparasitoids on parasitoid populations. Our results suggest that it would be feasible to implement biocontrol methods for aphids in integrated pest management programmes in peach orchards. There are a number of primary parasitoid species associated with these aphids, and the nearby crops and wild vegetation in the vicinity and within the orchards may provide a suitable habitat for them. Additionally, some of them are commercially available and might be usable in augmentative releases.

## 1. Introduction

Peaches and nectarines (*Prunus persica* L. Batsch) are economically important crops worldwide. China is the world’s leading producer, with more than 50% of global peach crops by both area and yield, followed by Europe and North America. Within Europe, Spain is a leading producer and accounts for more than 30% of total European production [1], and most of Spain’s peach orchards are concentrated in the Catalonia region in northeastern Spain [2]. Ten aphid species have been reported to infest European peach trees [3,4], with the green peach aphid, *Myzus persicae* (Sulzer) and species of the *Hyalopterus* genus being the most serious pests. Peach trees are the primary host for both aphids and are necessary for their survival [5,6]. Although to our knowledge there has been no formal evaluation of yield loss due to these pests [7], they have been acknowledged as causing important damage to peach crops, including leaf twisting and pitting and discoloration of fruits. They are also important vectors of the plum pox virus or sharka, which is a serious disease affecting stone fruits [8,9,10].

To date, aphid control in most crops has mainly involved the use of insecticides, but there is increasing concern about their use and the risk they pose to both humans and the environment [7]. In addition, resistance to a wide range of insecticides, such as pyrethroids, organophosphates and carbamates has been recorded [10,11]. In the northeastern region of Spain, *M. persicae* have also been reported to have developed resistance to neonicotinoids [12]. Alternative tools for managing aphids are therefore urgently needed, and biological control could be a useful component of integrated pest management (IPM) programmes.

Parasitoids are among the main aphid biocontrol agents and several species have been shown to be effective in reducing aphid populations [10,13,14,15]. Hyperparasitoids, on the other hand, attack parasitized aphids, and therefore, present a risk to the short-term biological control of aphids [16,17,18,19,20]. It has been predicted that an equilibrated system that includes host aphids, primary parasitoids and hyperparasitoids may be beneficial for long-term biological control in the agroecosystem [21].

As a first step in designing a biocontrol-based IPM programmes to control aphids in peach orchards, it is crucial to identify the species most abundantly involved in the parasitoid-hyperparasitoid system. This information will be useful not only for promoting conservation biological control but also for selecting the most convenient parasitoid species to release, if augmentative releases to complement natural biological control are determined to be necessary. Therefore, the present study was undertaken to identify naturally occurring hymenopteran species and their relative abundance on *M. persicae* and *Hyalopterus* spp., the aphid species that cause the most damage to peach trees.

## 2. Materials and Methods

Samples were collected from two areas within Catalonia: one area was located inland (Lleida), and the other was in the coastal area of Barcelona. Lleida is one of the biggest peach and nectarine production areas in Spain, accounting for 24% of total Spanish production, and it has almost 20,000 hectares of peach and nectarine orchards. In contrast, the Barcelona area has only 587 hectares [2]. Sample sites in Lleida consisted of four commercial organic orchards with the following GPS coordinates: 41.718, 0.618 in Vilanova de Segrià, 41.627, 0.541 in Torres de Sanui, 41.810, 0.582 in Almenar and 41.832, 0.548 in Alfarràs. In Barcelona, the survey was conducted in one experimental plot at IRTA facilities located at 41.516, 2.372 in Cabrils, with hybrid trees derived from an initial cross of *P. persica* × *P. dulcis*. The study was conducted between April 2015 and July 2017.

### 2.1. Species Identification of M. persicae Parasitoids and Hyperparasitoids

The Lleida sites were sampled using two methods: sentinel plants and random collections of aphid-infested shoots (Table 1). Sentinel plants consisted of small potted peach plants (approximately 50 cm tall) infested with *M. persicae*. To infest the plants, excised peach buds with approximately 100 *M. persicae* (adults and different instars), collected from the same orchards in Lleida, were placed on top of the plants and kept there for one week before taking the plants to the field. During this time, the plants were kept in a closed screened greenhouse to prevent contamination. Once in the orchards, 16 sentinel plants per field were placed under the peach tree canopy: 4 sentinels were placed near the first tree of 4 adjacent rows and another 4 sentinels were placed between the second and the third trees of the same rows. Another 8 sentinels were placed following the same pattern at the other end of the field. The distance between the two groups of 8 plants differed depending on the size of the field and ranged from 25 to 130 m. The pots containing the sentinel plants were placed inside bigger pots with water to avoid desiccation, and the outside of the outer pots was sprayed with insect-trapping adhesive to prevent ants and other soil predators from climbing the plant. Sentinel plants were taken to the orchards at 15-day intervals and were left there for one week. Then, the infested leaves were collected and taken to the laboratory. The sampling period lasted from mid-April to the end of June, which is the period when *M. persicae* populations typically damage crops in the sampled area.

To complement the sampling with sentinel plants, random samples of naturally-infested shoots were also collected from the same orchards during the same sampling periods. Neither the number of samples nor the periodicity of this sampling followed a specific pattern, because the number of infested trees varied between orchards and sampling dates (Table 1).

All plant samples from both the sentinel plants and the random sampling were transported to the laboratory in ice chests. Once in the laboratory, predators were removed from samples and the aphid colonies were placed in mesh-covered, semi-transparent plastic boxes in climatic chambers at 25 °C that were checked daily during working days until either parasitoids or hyperparasitoids emerged. For the sentinel plants, each plant was processed individually as a single sample. For the random collection samples, all infested twigs from one date and one field were pooled and treated as a single sample. Adult parasitoids and hyperparasitoids were preserved in 70% alcohol in microtubes (2 mL), and individuals were classified at the species level using taxonomic keys by Graham [22], Kamijo and Takada [23], Rakshani et al. [24,25], Kavallieratos et al. [26], and Ghaliow et al. [27]. All samples belonging to the *Encyrtidae* family were sent to Dr John Noyes at the British Museum for further classification. Species were identified as either primary parasitoids or hyperparasitoids following the classification by Sullivan [20].

### 2.2. Species Identification of Hyalopterus spp. Parasitoids and Hyperparasitoids

We collected random samples of peach shoots infested by *Hyalopterus* spp. in Lleida and Barcelona sites from mid-April to the end of July (Table 1). As above, neither the number of samples nor the periodicity of this sampling followed a specific pattern, because the number of infested trees varied between orchards and sampling dates (Table 1). Samples were handled using the same methodology described above for the parasitoids and hyperparasitoids of *M. persicae*.

## 3. Results

### 3.1. Species Identification of M. persicae Parasitoids and Hyperparasitoids

A total of 626 parasitoids and 57 hyperparasitoids were collected from the sentinel plants infested with *M. persicae* during two years of sampling, while 246 parasitoids and 124 hyperparasitoids were collected from the randomly collected samples over three years of sampling. We identified a total of 11 different *M. persicae* primary parasitoid species from six genera. Of these 11 species, *Aphidius matricariae* Haliday (Hymenoptera: Braconidae: Aphidiinae) was by far the most abundant in all three years and within both the sentinel plants and the randomly collected samples. This species accounted for 91% of the primary parasitoids that emerged from aphid mummies (Table 2).

Within the same samples, we also identified 10 species of hyperparasitoids associated with *M. persicae.* Six of these species belonged to the *Figitidae* family, three belonged to the *Pteromalidae* family and one belonged to the *Encyrtidae* family (Table 2). The most abundant species from both sampling methods was *Phaenoglyphis villosa* (Hartig) (Hymenoptera: Figitidae), which ranged from 20% of the hyperparasitoids collected from the sentinel plants in 2015 to almost 70% in 2016, as well as 20% of the hyperparasitoids collected from the pooled random samples across the whole 3-year period. *Asaphes suspensus* (Nees) (Hymenoptera: Pteromalidae) was the second-most abundant species; however, it was only found in the random samples. 

**Figure 1:**
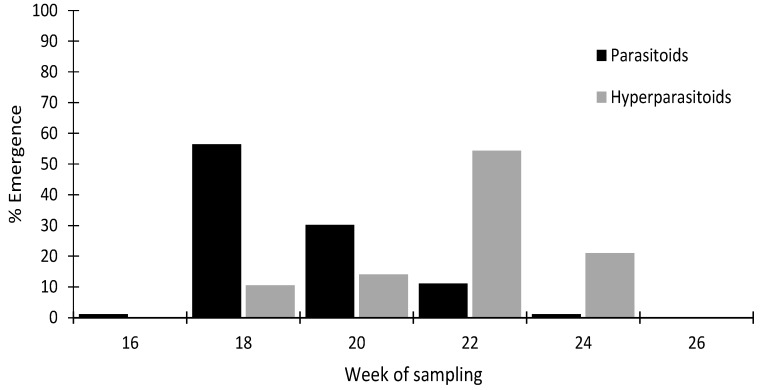
Bi-weekly seasonality of *M. persicae* parasitoids and hyperparasitoids from sentinel plants. The percentages of parasitoids and hyperparasitoids per sampling date were calculated based on the total number of parasitoids and hyperparasitoids, respectively, that emerged from aphid mummies.

Seasonal abundance differed between the primary parasitoids and the hyperparasitoids collected from the sentinel plants (Figure 1). The majority of the primary parasitoids were collected in week 18 (late April), whereas the majority of the hyperparasitoids were collected in week 22 (late May).

### 3.2. Species Identification of Hyalopterus spp. Parasitoids and Hyperparasitoids

Out of the total of 558 parasitoids collected from *Hyalopterus* spp. samples, we identified only two different species, *Aphidius transcaspicus* Telenga and *Praon volucre* (Haliday) (Hymenoptera: Braconidae: Aphidiinae), during the three years of the study across both areas. Surprisingly, 82% of all parasitoids collected were *A. transcaspicus* specimens collected from a single sample in Barcelona. Among the rest of the collected samples, *A. transcaspicus* and *P. volucre* comprised 46% and 54%, respectively.

**Figure 2:**
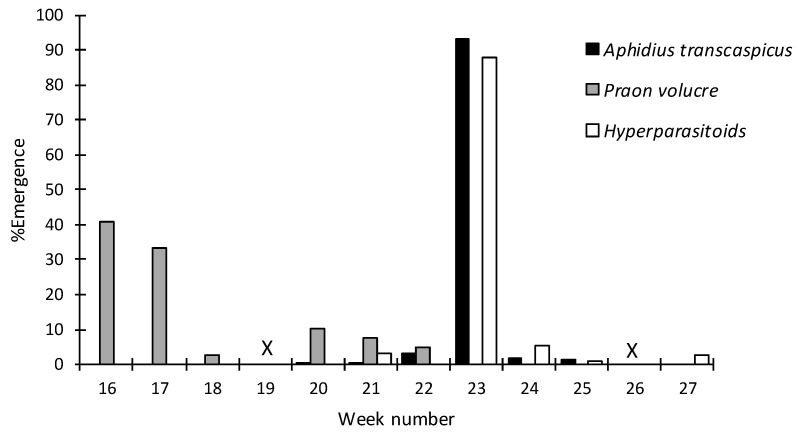
Seasonality of the *Hyalopterus* spp. parasitoid and associated hyperparasitoid species collected in Barcelona in 2017. The percentages of parasitoids and hyperparasitoids per sampling date were calculated from the total number of parasitoids and hyperparasitoids, respectively, that emerged from the mummies. X indicates that no sampling was conducted during that week.

The most intensive sampling efforts for *Hyalopterus* spp. aphids occurred in 2017 in the Barcelona site; Figure 2 shows the seasonality of *Hyalopterus* spp. parasitoids for this year in this experimental plot. *Aphidius transcaspicus* was recorded from mid-May until mid-June, with 93% of all *A. transcaspicus* specimens collected at the beginning of June (week 23). Conversely, *P. volucre* individuals were collected from the beginning of sampling in mid-April (week 16) until the end of May (week 22), with the highest numbers being collected in weeks 16 and 17.

We collected 394 hyperparasitoids associated with *Hyalopterus* spp. during the 3-year study, including both surveyed areas. These were comprised of five different species of hyperparasitoid, with two belonging to the *Figitidae* family, one to the *Encyrtidae* family, and two to the *Pteromalidae* family. In both locations, the most abundant *Hyalopterus* spp. associated hyperparasitoid species was *Pachyneuron aphidis* (Bouché) (Hymenoptera: Pteromalidae: Pteromalinae) (Table 3), which accounted for 87% of all *Hyalopterus* spp. associated hyperparasitoids. *Syrphophagus nr. africanus* was the second-most abundant *Hyalopterus* spp. associated hyperparasitoid but accounted for only 5% of the total, and was found mainly in the Lleida samples.

## 4. Discussion

During our 3-year study on peach trees, the most abundant *M. persicae* primary parasitoid species was, by far, *A. matricariae*, which accounted for 91% of all *M. persicae* parasitoids collected in our study. The tritrophic association between *P. persica*, *M. persicae*, and *A. matricariae* has been previously recorded in other regions of Spain [28], and *A. matricariae* have in fact long been recognised as the most common and probably the most effective parasitoids of *M. persicae* [29]. This tritrophic association has also been found in other European countries, such as France [30,31], Greece and Serbia [4].

However, while surveys by Michelena et al. [32,33] found only two species of *M. persicae* parasitoids on peach trees, our study identified 11 different species. Six of these have been previously reported on *M. persicae* on peach trees in other European countries, namely *Aphidius colemani* Viereck, *Aphidius ervi* Haliday, *Ephedrus persicae* Froggat, *P. volucre, Diaeretiella rapae* (McIntosh) and *Lysiphlebus testaceipes* (Cresson) (Hymenoptera: Braconidae: Aphidiinae) [4,30,31,34,35]. However, our sampling identified three additional tritrophic associations that have not previously been reported in Europe: *A. transcaspicus, Ephedrus plagiator* (Nees) *and Praon abjectum* (Haliday) (Hymenoptera: Braconidae: Aphidiinae). 

Given that *P. persica* trees are the primary hosts of *M. persicae*, parasitoids in early spring have to come from other surrounding crops. Boivin et al. [36] emphasised the importance of suitable reservoirs near or within fields (e.g., in grassy ground covers in orchard alleys) for the survival of aphid parasitoids. In the Lleida study area, our field sites were close to other orchards and arable crops, (e.g., wheat, barley, maize, alfalfa, oats and rye grass). Pons et al. [37] and Lumbierres et al. [28] found *A. matricariae* to also be the predominant species of parasitoid of several aphid species (*Rhopalosiphum padi* (L.), *Rhopalosiphum maidis* (Fitch), *Samilyitobion avenae* (Fabricious) and *Metopolophium dirhodum* (Walker)] (Hemiptera: Aphidiidae) that infest such crops. Aphids in nearby crop fields may therefore act as winter reservoirs for *A. matricariae* before they move to orchards in spring.

Although much less abundant (3.3% of total parasitoids), *A. ervi* was nonetheless the second-most prevalent parasitoid of *M. persicae* in our study. Interestingly, high rates (≥80%) of *A. ervi* have been recorded by Pons and Starý [38] on *Acyrthosiphon pisum* (Harris) (Hemiptera: Aphidiidae) on alfalfa and on *S. avenae* aphids on wheat during spring in the same area where our sampling took place. The differences between our results and theirs may be because *A. ervi* is an oligophagous species that is most commonly found parasitising cereal aphids [39], and *M. persicae* are probably not among their preferred hosts. Also of note is that *A. ervi* has among the highest market values of any aphid parasitoids sold worldwide for aphid control [40].

*Lipolexis* sp. and *A. colemani* were the next two most prevalent species of parasitoids of *M. persicae* in our samples. Only two species of *Lipolexis* sp. have been recorded in Europe, namely, *L. gracilis* Förster and *L. oregmae* Gahan. However, significant confusion exists in the systematics and taxonomy of this genus, suggesting that several cryptic species could be hidden [41], and additional studies are therefore needed to identify our specimens at the species level. *Lipolexis* spp. are commonly found parasitising species of aphids belonging to the genera *Brachycaudus*, *Hyalopterus* and *Myzus*, the latter two of which are common in peach crops [4,30,31,42,43,44,45]. However, according to Mackauer [29], *Lipolexis* spp. show a marked preference for *Brachycaudus* species, which are less prevalent in stone-fruit trees than *M. persicae* and *Hyalopterus* spp. [8]. *Aphidius colemani*, meanwhile, is presumed to be native to India but has become accidentally widespread in many areas of the world, including Mediterranean Europe [46]. This species has also been widely used as a biocontrol agent in greenhouses [40], and its establishment outside of its area of origin may be a consequence of accidental escapes from these confined environments [47,48].

In our surveys of *M. persicae*, we identified minor numbers of seven more species (see Table 2). Of these, *A. transcaspicus, E. plagiator* and *P. abjectum* are known to parasitise other aphid species that are common in stone and pip fruit trees in the Mediterranean [4,35,42,43,44,45,46,49,50,51,52]. According to Kavallieratos et al. [53], *A. transcaspicus* is highly specific to *Hyalopterus* spp. The occurrence of this parasitoid on *M. persicae* in our field samples may simply be fortuitous, since it was registered on only one date during the first sampling year. However, Wang and Messing [54] found that this parasitoid successfully attacked *M. persicae* under laboratory conditions. Notably, *A. transcaspicus* was also one of only two species that were recruited from our *Hyalopterus* spp. samples. It also has been shown to be an effective natural enemy of *Hyalopterus* spp. on peach trees in other European countries [55,56].

In our surveys of *Hyalopterus* spp., in addition to *A. transcaspicus* we also identified *P. volucre*. This parasitoid has been previously recorded in Spain by González and Michelena [57] in peach trees infested with *Hyalopterus* spp. Pons and Starý [38] also found this parasitoid on *Hyalopterus amygdali* Blanch (Hemiptera: Aphididae) infesting cherry trees (*Prunus avium* (L.)) in the Lleida area, as well as on several aphid species infesting nearby maize. Furthermore, Starý [58] and Tomanović and Brajkovic [59] have also noted that this parasitoid is commonly found on cereal aphids. It has also been found on aphids infesting wild vegetation, including species from the *Poaeceae* family. This wider host range may explain their appearance early in the season, since its population increase is not dependent on *Hyalopterus* spp. colonies.

We also found that *M. persicae* and *Hyalopterus* spp. had overlapping hyperparasitoid complexes, and hyperparasitoid species belonging to the Pteromalidae, Encyrtidae and Figitidae families were observed on both parasitoids. However, their relative abundance differed between *M. persicae* and *Hyalopterus* spp. In addition, all but one of the species identified in our samples have been previously recorded in Spain [60,61,62,63], but to the best of our knowledge, ours is the first report of *S. nr*. *africanus* in Spain or elsewhere in Europe.

The hyperparasitoid species composition on our *M. persicae* samples varied according to the survey methodology used. Our sentinel plants yielded only one *P. aphidis* individual and none belonging to either species of the *Asaphes* genus, whereas these three species were abundant in our random samples of plants infested with *M. persicae* aphids. This may be due to hyperparasitoid biology. *Pachyneuron aphidis*, *A. suspensus* and *A. vulgaris* are ectophagous idiobionts that parasitise only mummified aphids [20]. Our sentinel plants were brought to the field with only live aphids and were exposed for only one week, which is too short a period of time for mummies to have been formed [64]. Consistent with this explanation, in the samples from sentinel plants the identified hyperparasitoids were almost exclusively endophagous koinobiont species—i.e., species that attack live aphids [20,65]. *Syrphophagus* nr. *africanus*, which can parasitise both live aphids and mummies [66], was collected with both sampling methods. The results from the random samplings are therefore likely to better reflect the hyperparasitoid complexes that attack *M. persicae*’s and *Hyalopterus* spp.’s primary parasitoids in peach orchards.

## 5. Conclusions

Our study found that a wide array of primary parasitoids associated with *M. persicae* were present in *P. persica* orchards. The prevalence of this array of parasitoids should make the ecosystem more stable and resilient to potential invasions of new aphid species [3,20,36]. *Aphidius matricariae* accounted for over 90% of all our primary parasitoids, suggesting that it should be the key parasitoid species considered as a biological control agent in conservation biological control programs and, probably the best candidate for augmentative releases. Moreover, it is commercially available and is widely used in greenhouse crops for aphid control [67]. In contrast, we found only two parasitoids associated with *Hyalopterus* spp. aphids: *A. transcaspicus* and *P. volucre*. However, either of these could likely function as biological control agents of *Hyalopterus* spp. aphids.

In addition, given that *A. matricariae* and *P. volucre* are commonly found in the arable crops surrounding the peach orchards in Lleida area, these crops may represent an important source of parasitoids. Spontaneous flora within and near the orchards may be also important for these parasitoids’ survival, by providing them with alternative hosts, food and refuge, particularly during winter [68,69,70,71,72]. Consistent with this theory, laboratory experiments have confirmed that *Aphidinae* improve their fitness when flowers are available [73,74].

Additionally, if future surveys confirm our finding of a spontaneous association between *A. transcaspicus* and *M. persicae* at increasing numbers, this may allow for improved biological control of *Hyalopterus* spp. *Myzus persicae* usually infest peach trees earlier in the season than do *Hyalopterus* spp. [8,75]; therefore, increased parasitism on *M. persicae* early in the peach season might contribute to increased parasitoid populations prior to the heavy infestations of *Hyalopterus* spp. that damage trees.

Although hyperparasitoids might be beneficial to the long-term stabilisation of insect- parasitoid dynamics [20,76], in the short term they may be detrimental to aphid control. The absence of hyperparasitoids early in the season, when *M. persicae* populations build up, suggest that biological control of this aphid would not be negatively impacted by the presence of hyperparasitoids. However, this would not be the case for *Hyalopterus* spp. whose populations peak later in the season and, therefore, its control would be negatively affected by the presence of hyperparasitoids, which are more abundant at this time of year. Overall, our results suggest that biological control of aphids in peach trees is feasible and should be considered for IPM programmes; however, strategies to boost parasitoid populations should probably be adopted.

## Figures and Tables

**Table 1:** Sampling conducted to identify the parasitoid and hyperparasitoid species associated with *M. persicae* and *Hyalopterus* spp. and their abundance in peach trees. The time period of the samples collected is expressed in week numbers, where week 16 corresponds to mid-April and week 31 to end of July.

Aphid Species	Area	Sampling Method	Year		
			2015	2016	2017
			Number of Samples Collected (Time Period)		
*Myzus persicae*	Lleida	Sentinel plants	4 (18–24)	6 (16–26)	—
		Random samples	5 (18–27)	6 (17–23)	2 (19–20)
*Hyalopterus spp.*	Lleida	Random samples	5 (22–31)	2 (23–28)	1 (21)
	Barcelona	Random samples	—	3 (23–26)	10 (16–27)

**Table 2:** *Myzus persicae* parasitoids and hyperparasitoids, collected on peach trees using both sampling methods during the 3-year study.

Family and Subfamily	Species	Sentinel Plants		Random Sampling		
		2015	2016	2015	2016	2017
Parasitoids						
Braconidae Aphidiinae						
	*Aphidius matricariae* (Haliday)	344	213	27	26	180
	*Aphidius ervi* Haliday	12	8	0	0	9
	*Aphidius colemani* Viereck	1	16	0	0	0
	*Aphidius transcaspicus* Telenga	2	0	0	0	0
	*Lipolexis sp.*	4	11	0	0	1
	*Ephedrus persicae* Froggat	0	5	0	3	0
	*Ephedrus plagiator* (Nees)	1	0	0	0	0
	*Praon volucre* (Haliday)	1	5	0	0	0
	*Praon abjectum* (Haliday)	0	1	0	0	0
	*Diaeretiella rapae* (McIntosh)	1	0	0	0	0
	*Lysiphlebus testaceipes* (Cresson)	0	1	0	0	0
Hyperparasitoids						
Figitidae Charipinae						
	*Phaenoglyphis villosa* (Hartig)	6	18	1	23	0
	*Alloxysta pusilla* (Kieffer)	3	4	2	14	0
	*Alloxysta victrix* (Westwood)	0	5	0	3	0
	*Alloxysta arcuata* (Kieffer)	4	0	0	0	4
	*Alloxysta castanea* (Hartig)	0	0	0	1	0
	*Alloxysta fuscicornis* (Hartig)	0	0	0	0	1
Pteromalidae Asaphinae						
	*Asaphes suspensus* (Nees)	0	0	0	38	1
	*Asaphes vulgaris* (Walker)	0	0	0	4	0
Pteromalinae						
	*Pachyneuron aphidis* (Bouché)	1	0	0	13	10
Encyrtidae Encyrtinae						
	*Syrphophagus nr. africanus*	16	0	0	9	0

**Table 3:** Number of *Hyalopterus* spp. associated hyperparasitoid species collected during the 3-year study in both surveyed areas.

Families and Subfamilies	Species	Lleida	Barcelona
Pteromalidae			
Pteromalinae	*Pachyneuron aphidis* (Bouche)	53	260
Asaphinae	*Asaphes suspensus* (Nees)	2	8
Encyrtidae			
Encyrtinae	*Syrphophagus nr. africanus*	19	3
Figitidae			
Charipinae	*Phaenoglyphis villosa* (Hartig)	1	2
	*Alloxysta fuscicornis* (Hartig)	1	2
